# Supplementary material for: RNA Sequencing Reveals the Potential Adaptation Mechanism to Different Hosts of Grapholita molesta
Source: Insects. 2022 Sep 30;13(10):893. doi: 10.3390/insects13100893 (PMC9604371; doi:10.3390/insects13100893)

RNA-sequencing reveals the potential adaptation mechanism to different hosts of *Grapholita molesta*

Dongbiao Lü [1], Zizheng Yan [1], Di Hu [1], Aiping Zhao [1], Shujun Wei [2], Ping Wang [3], Xiangqun Yuan [1*] and Yiping Li [1*]

1. Key Laboratory of Integrated Pest Management on Crops in Northwestern Loess Plateau, Ministry of Agriculture, College of Plant Protection, Northwest A&F University, Yangling, Shaanxi 712100, China.

2. Institute of Plant Protection, Beijing Academy of Agriculture and Forestry Sciences, 9 Shuguanghuayuan Middle Road, Haidian District, Beijing, 100097, China.

3. Department of Entomology, Cornell University, Geneva, NY, 14456, USA.

* Correspondence: yuanxq@nwsuaf.edu.cn, liyiping@nwsuaf.edu.cn.

Table legends

Table S1 Analysis of the difference of FPKM and expression in feeding on different hosts by *G. molesta*.

Table S2 Primers used for quantitative real-time PCR (qRT-PCR).

Table S3 Summary of sequencing for the Illumina operation of the experimental samples.

Table S4 Sample and reference genome mapping statistics.

Figure legends

Figure S1 FPKM density distribution of each sample. The X-coordinate is log2 (FPKM+1), and the Y-coordinate is gene density.

Figure S2 FPKM distribution violin of each sample. The X-coordinate is the sample name, and the Y-coordinate is log2 (FPKM+1).

Figure S3 Heat map of Pearson correlation between samples.

Figure S4 Up-regulated GO enrichment pathways of DEGs pairwise of AD vs. PL.

Figure S5 Up-regulated GO enrichment pathways of DEGs pairwise of PR vs. PL.

Figure S6 Down-regulated GO enrichment pathways of DEGs pairwise of PL vs. PS.

Figure S7 Down-regulated GO enrichment pathways of DEGs pairwise of PR vs. PS.

Figure S8 Soft threshold selection requirements. The horizontal axis of the graph all represent the weight parameter β, which is the soft threshold. The vertical axis of the left figure represents the square of the correlation coefficient in the corresponding network. The vertical axis of the figure on the right represents the mean of all gene adjacency functions in the corresponding gene module.

Table S1 Analysis of the difference of FPKM and expression in feeding on different hosts by *G. molesta*

Table S2 Primers used for quantitative real-time PCR (qRT-PCR)

**Target genes**	**Direction**	**Sequence 5' to 3'**
gm_26520-RA	F	GCTTCTTGTTGCGTTTGCCTT
	R	CGATCTCCACCACCGAGT
gm_38295-RA	F	ACTTCGATGGAGCCAGGA
	R	AGTAGCCCCATTGGTGAGAGT
gm_04701-RA	F	AGTCCGAGGCCATGAACT
	F	ATAACAGTGGGCAGCAGTCAG
novel.798	R	TATAGTCGGTGGCTGGGA
	F	CGGTTGCATAGTTTATCACACACG
gm_11542-RA	R	ATGACCTGCCCCATAACGA
	F	TGACGTACCAGACCTCGACT
gm_30636-RA	R	TCTCTCGACCTTCAGGACAAC
	F	TGATGCGTCCCAGCCAC
gm_06101-RA	R	CAACGAAAACCGTCGCCTC
	F	GAGAAAACCACCAGCTTGCC
gm_35262-RB	R	CCCTACTACTTGTGTGACGACCA
	F	CTTTGCCGAACCTGATGTCC
gm_35484-RH	R	CGACGCATCATAAAACATCCAT
	F	AGTCCAGTTTGATCGGATTCTCA
β-Action	R	TGCGTGACATCAAGGAGAAG
	F	TACCGATGGTGATGACCTGA
EF-1α	R	AGGAGATCGAGCAACAGGAA
	F	CACGACTCTCGGGACTTCTC

Table S3 Summary of sequencing for the Illumina operation of the experimental samples

Sample	Raw reads	Clean reads	Clean percent	Error rate	Q20	Q30	GC
			(%)				
AD1	46459020	42145670	90.72	0.02	95.02	88.35	48.09
AD2	47109588	42976246	91.23	0.02	95.31	88.87	46.59
AD3	49892310	45318172	90.83	0.02	95.6	89.51	47.60
PL1	56088982	54446956	97.07	0.03	97.19	92.84	53.69
PL2	54286856	52633444	96.95	0.03	97.24	92.81	51.47
PL3	47693870	46455168	97.40	0.03	97.42	93.18	51.63
PS1	38070174	36448254	95.74	0.02	98.49	95.66	57.25
PS2	47706866	46696850	97.88	0.02	98.32	95.01	53.55
PS3	49082110	47740992	97.27	0.02	98.28	94.92	54.73
AP1	47057454	45692854	97.10	0.02	98.07	94.66	57.98
AP2	50287024	49260108	97.96	0.02	98.2	94.85	56.10
AP3	46646934	45807938	98.20	0.02	98.86	96.34	56.96
PC1	48265980	47391492	98.19	0.02	98.13	94.69	52.61
PC2	61963606	60618820	97.83	0.02	98.12	94.67	52.26
PC3	55246116	54192148	98.09	0.02	97.88	94.33	55.07
PR1	47708442	46571318	97.62	0.02	98.03	94.45	52.13
PR2	51013002	49822584	97.67	0.02	98.05	94.55	52.36
PR3	46431674	45342746	97.65	0.03	97.89	94.27	53.05

Table S4 Sample and reference genome mapping statistics

Sample	Total reads	Total map	Unique map	Read1 map	Read2 map	Positive map	Negative map	Proper map
AD1	42145670	33394006 (79.23%)	32156084 (76.30%)	16371149 (38.84%)	15784935 (37.45%)	15977198 (37.91%)	16178886 (38.39%)	28807722 (68.35%)
AD2	42976246	33351588 (77.60%)	32041343 (74.56%)	16299351 (37.93%)	15741992 (36.63%)	15966164 (37.15%)	16075179 (37.40%)	28583688 (66.51%)
AD3	45318172	36356295 (80.22%)	34934256 (77.09%)	17740039 (39.15%)	17194217 (37.94%)	17359768 (38.31%)	17574488 (38.78%)	31461374 (69.42%)
PL1	54446956	45675313 (83.89%)	43591906 (80.06%)	21772485 (39.99%)	21819421 (40.07%)	21733953 (39.92%)	21857953 (40.15%)	38730258 (71.13%)
PL2	52633444	42387590 (80.53%)	40595020 (77.13%)	20269769 (38.51%)	20325251 (38.62%)	20238044 (38.45%)	20356976 (38.68%)	35897814 (68.20%)
PL3	46455168	38461940 (82.79%)	37021481 (79.69%)	18480862 (39.78%)	18540619 (39.91%)	18467147 (39.75%)	18554334 (39.94%)	33262964 (71.60%)
PS1	36448254	28117963 (77.14%)	26821779 (73.59%)	13409167 (36.79%)	13412612 (36.8%)	13476164 (36.97%)	13345615 (36.62%)	24387484 (66.91%)
PS2	46696850	37345852 (79.98%)	35699728 (76.45%)	17835797 (38.19%)	17863931 (38.26%)	17840526 (38.2%)	17859202 (38.24%)	32688682 (70.00%)
PS3	47740992	39984690 (83.75%)	38180419 (79.97%)	19103207 (40.01%)	19077212 (39.96%)	19142690 (40.1%)	19037729 (39.88%)	35113886 (73.55%)
AP1	45692854	33594432 (73.52%)	31543961 (69.03%)	15790389 (34.56%)	15753572 (34.48%)	15880278 (34.75%)	15663683 (34.28%)	28034058 (61.35%)
AP2	49260108	38086096 (77.32%)	36363857 (73.82%)	18199253 (36.95%)	18164604 (36.87%)	18220524 (36.99%)	18143333 (36.83%)	33583982 (68.18%)
AP3	45807938	37374764 (81.59%)	36064508 (78.73%)	18027858 (39.36%)	18036650 (39.37%)	18053606 (39.41%)	18010902 (39.32%)	34037398 (74.30%)

PC1	47391492	42334608 (89.33%)	40605693 (85.68%)	20357645 (42.96%)	20248048 (42.73%)	20303963 (42.84%)	20301730 (42.84%)	37624216 (79.39%)
PC2	60618820	54046080 (89.16%)	51724909 (85.33%)	25928583 (42.77%)	25796326 (42.55%)	25891070 (42.71%)	25833839 (42.62%)	48090312 (79.33%)
PC3	54192148	46083617 (85.04%)	43596332 (80.45%)	21882006 (40.38%)	21714326 (40.07%)	21874784 (40.37%)	21721548 (40.08%)	39739416 (73.33%)
PR1	46571318	40166193 (86.25%)	38391813 (82.44%)	19254022 (41.34%)	19137791 (41.09%)	19225960 (41.28%)	19165853 (41.15%)	35363000 (75.93%)
PR2	49822584	42360094 (85.02%)	40510297 (81.31%)	20312102 (40.77%)	20198195 (40.54%)	20301715 (40.75%)	20208582 (40.56%)	37336132 (74.94%)
PR3	45342746	39177336 (86.40%)	37348382 (82.37%)	18745648 (41.34%)	18602734 (41.03%)	18708863 (41.26%)	18639519 (41.11%)	34182694 (75.39%)

Figure S1

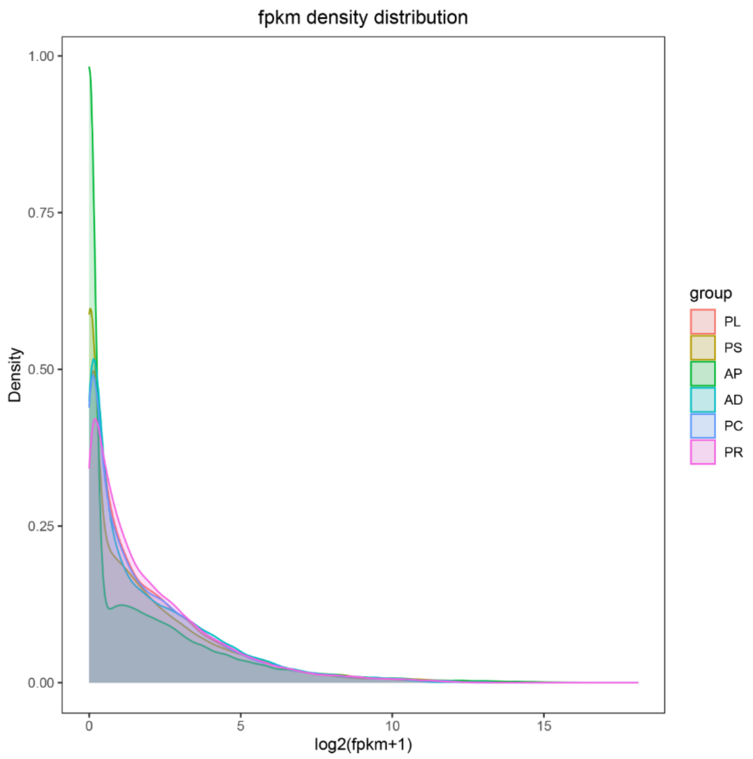

Figure S2

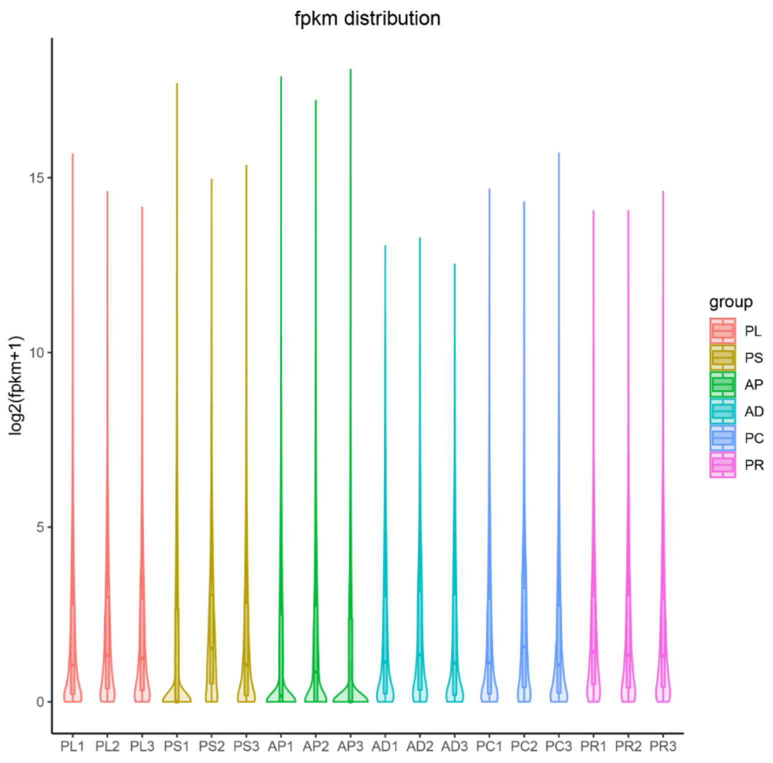

Figure S3

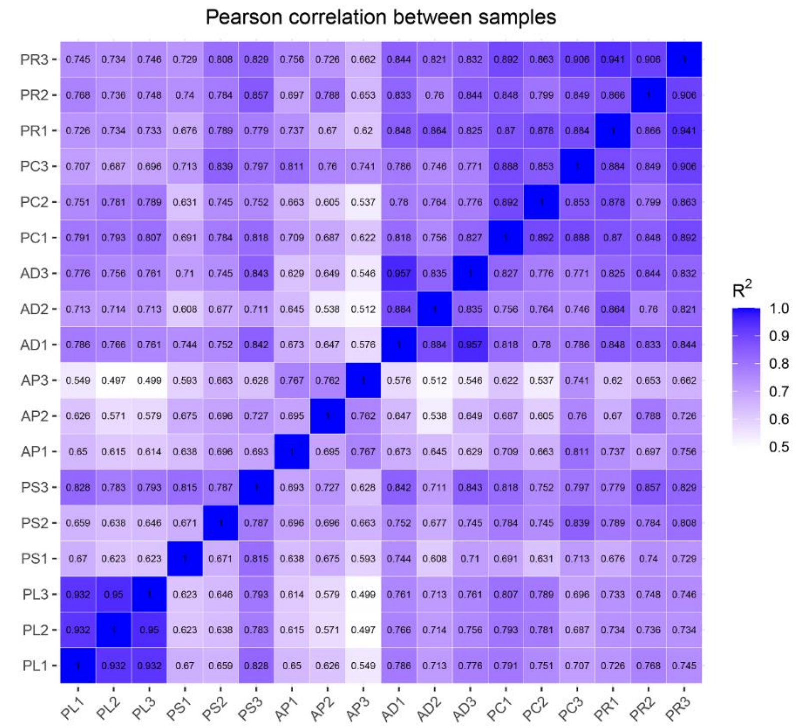

Figure S4

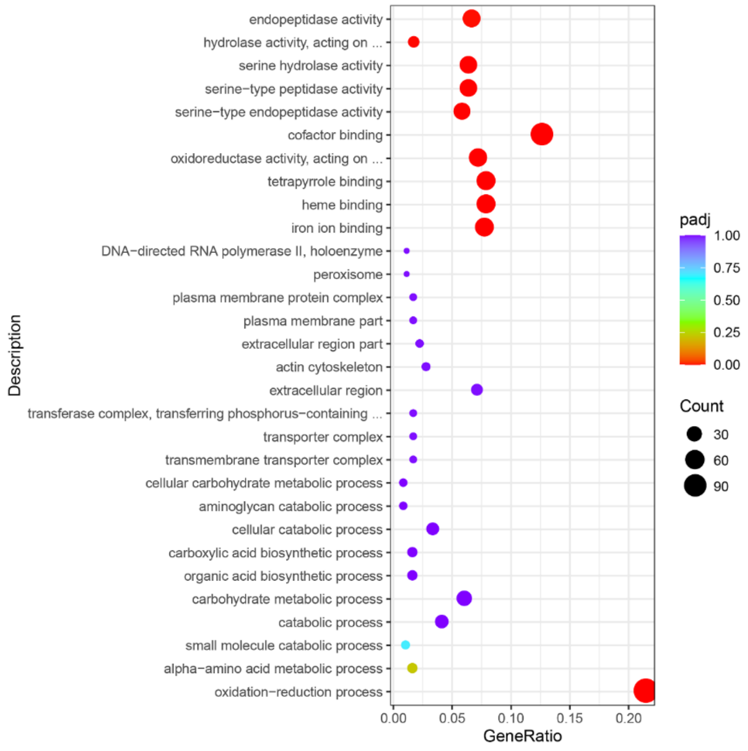

Figure S5

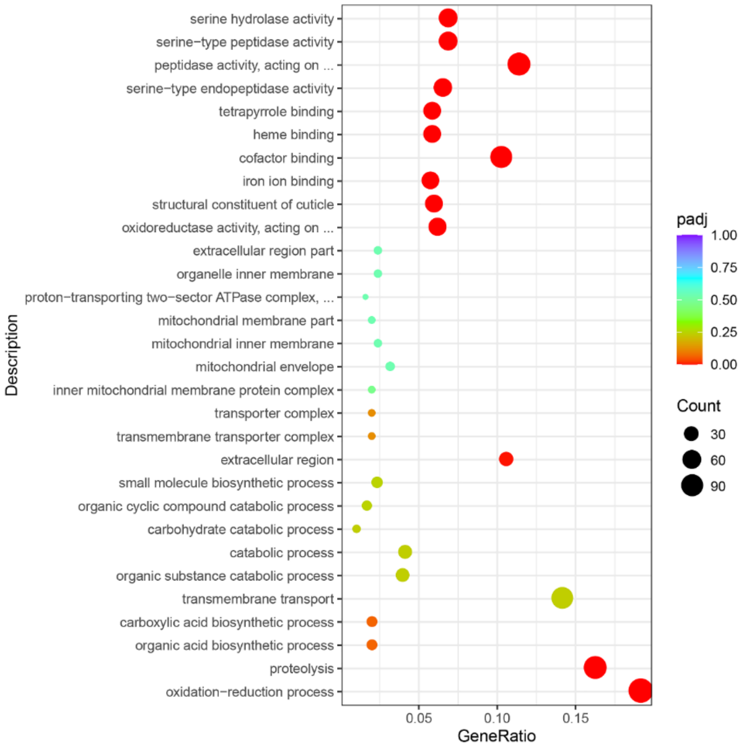

Figure S6

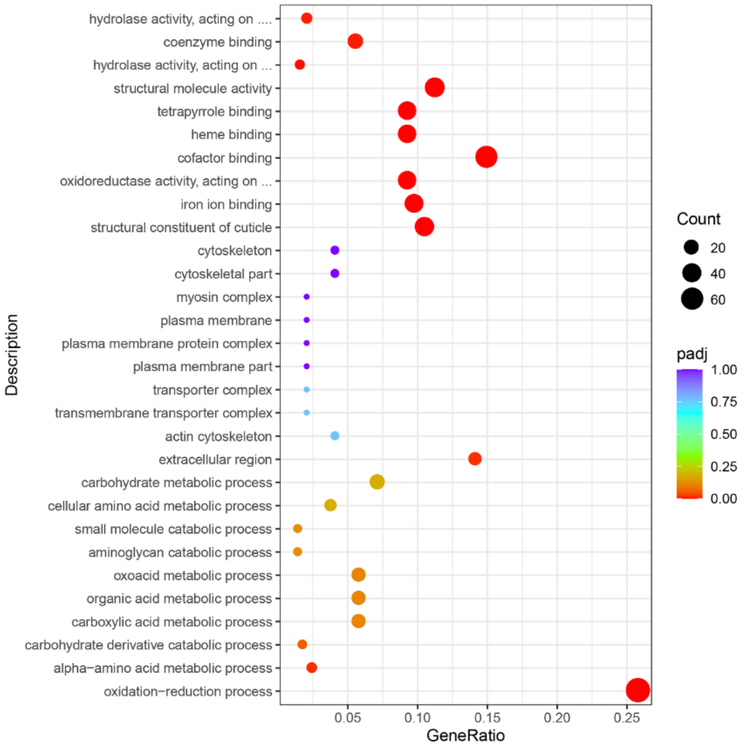

Figure S7

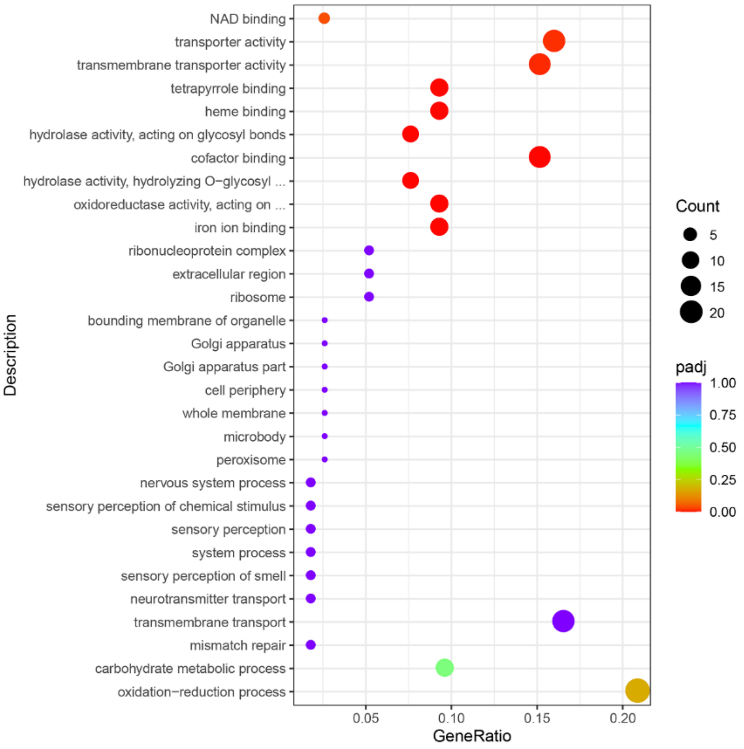

Figure S8

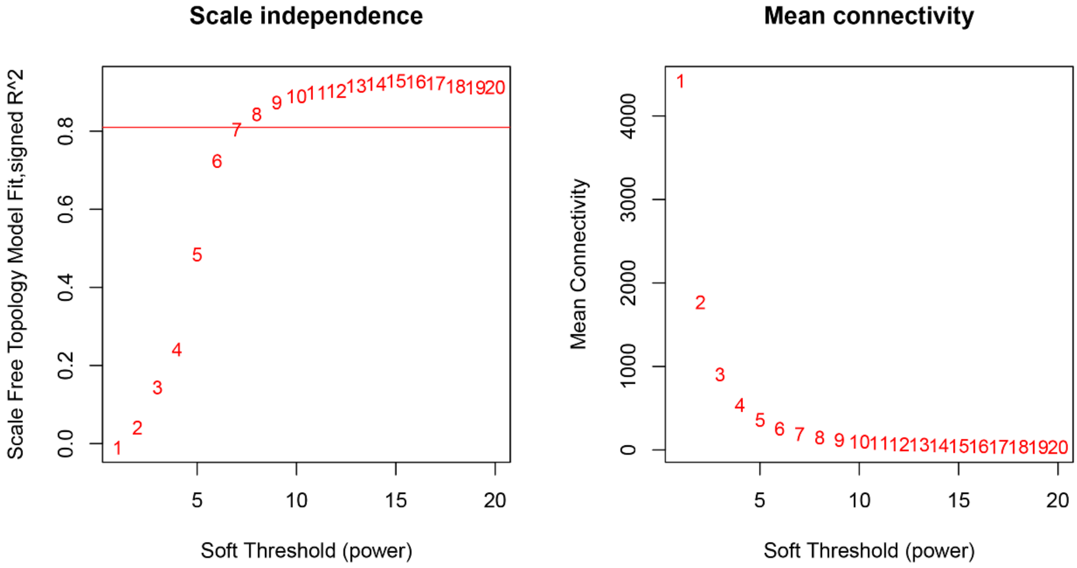

Supplement: Supplementary file 1 [file insects-13-00893-s001.zip › Support information.pdf]
